# Food allergy and anaphylaxis – 2040. Nutritional status and dietary intake in children with cow’s milk allergy

**DOI:** 10.1186/1939-4551-6-S1-P125

**Published:** 2013-04-23

**Authors:** Maria Golebiowska Wawrzyniak, Grazyna Rowicka, Malgorzata Strucinska, Katarzyna Markiewicz

**Affiliations:** 1 Department of Clinical Immunology, Institute of Mother and Child, Poland; 2 Department of Nutrition, Institute of Mother and Child, Poland

## Background

Poor different acceptance of milk-free formulas in the diet of children with allergy to cows’ milk (CMA) poses the risk of nutritional deficiencies especially calcium, vitamin D and iron and also can lead to malnutrition.

## Methods

Sixty children aged 2-5 years were divided into two groups: group I - 40 children with CMA, group II –20 healthy controls. In children diagnosed with allergy to cow’s milk, milk free diet was recommended.

Dietary intake and nutritional status were assessed at six-monthly intervals: at the beginning of the study and after 6 and 12 months of observation. Nutritional status of children was assessed with anthropometric traits and indices (i.e. Body Mass Index) and selected biochemical parameters were performed.

## Results

Despite of differences in the average concentration in the serum of children with both groups of proteins, albumin and iron, their values ranged of standards for age, as well as the assessed value of other biochemical parameters. At the beginning of the study BMI z-score of 75% of children in group I and 80% in group II ranged between -1,0 to +1,0 whereas BMI z-score in 25% of group I and 20% of group II between -2,0 and -1,0. After 12 month follow–up in 91,5 % of children of group I and 88% of group II BMI z-score was between -1,0 to +1,0 while in 8,5% of children in group I and 12% in group II between -2,0 to -1,0.

## Conclusions

Nutritional status of children with CMA assessed by body mass index, and selected biochemical tests was normal. In children during 12 month of period of the study, positive changes in dietary habits were observed.

Children with CMA should remain under pediatric and dietician care in order to monitor their nutritional status and diet.

Nutrition care is also indicated for children on a traditional diet.

